# Preferences for Text Messaging Supports During Youth Transition to Adult Mental Health Services: Theory-Informed Modified e-Delphi Study

**DOI:** 10.2196/51690

**Published:** 2024-08-27

**Authors:** Negar Vakili, Janet A Curran, Roisin Walls, Debbie Phillips, Alanna Miller, Christine Cassidy, Lori Wozney

**Affiliations:** 1 Centre for Research in Family Health IWK Health Halifax, NS Canada; 2 Strengthening Transitions in Care Lab IWK Health Halifax, NS Canada; 3 School of Nursing Dalhousie University Halifax, NS Canada; 4 Mental Health and Addictions Nova Scotia Health Halifax, NS Canada; 5 Mental Health and Addictions IWK Health Halifax, NS Canada; 6 Health Canada Montreal, QC Canada

**Keywords:** patient satisfaction, satisfaction, cross-sectional, survey, surveys, engagement, usage, technology use, transitional, transition, coordinated care, service, services, feature, features, need, needs, transitional care, information science, human-computer interaction, health behavior, text-messaging, messaging, text messages, text message, SMS, mental health, persuasive system design, youth, adolescent, adolescents, teen, teens, teenager, teenagers

## Abstract

**Background:**

For many young people, the transition from child to adult mental health services is a vulnerable time associated with treatment disengagement and illness progression. Providing service information and options to youth, appealing to them, and tailoring to their needs during this period could help overcome systematic barriers to a successful transition. We know little about how SMS text message–based interventions might be leveraged to support the motivational, informational, and behavioral needs of youth during this time. Ascertaining youth preferences for the content and functionality of an SMS text message service could inform prototype development.

**Objective:**

This study investigated consensus preferences among youth on important content, technology features, and engagement supports to inform a transition-focused SMS text message service.

**Methods:**

A modified e-Delphi survey design was used to collect demographics, current levels of technology use, importance ratings on message content, preferred technical features, and barriers and enablers to engagement for youth in Canada aged 16-26 years who have accessed mental health services within the past 5 years. Survey items on content were categorized according to the information-motivation-behavioral skills (IMB) model. Survey items on technical features were categorized according to the persuasive system design (PSD) model. A predefined consensus rating matrix and descriptive statistics were used to characterize the sample. The high consensus threshold was 70%.

**Results:**

A total of 100 participants, predominantly non-White (n=47, 47%), aged 20-26 years (n=59, 59%), and who had first accessed mental health services between the ages of 13 and 19 years (n=60, 60%), were selected. The majority (n=90, 90%) identified as daily SMS text message users. A high level of consensus on importance ratings was reported in 45% (9/20) of content items based on the IMB model. There were higher levels of consensus on importance ratings related to behavior domain items (3/3, 100%) than information domain items (4/9, 44%) or motivation domain items (2/8, 25%). A high level of consensus on importance ratings was reported in only 19% (4/21) of feature and functionality items based on the PSD model. Among PSD model categories, there was a high level of consensus on importance ratings in 8% (1/12) of the primary task support domain items and 100% (3/3) of the system credibility support domain items. None of the dialogue-support and social-support domain items met the high level of consensus thresholds. In total, 27% (27/100) of youth indicated that the most significant enabler for engaging with a transition-focused SMS text message intervention was the personalization of text messages.

**Conclusions:**

Scientists developing next-generation SMS text messaging interventions for this population need to consider how levels of consensus on different features may impact feasibility and personalization efforts. Youth can (and should) play an integral role in the development of these interventions.

## Introduction

Youth in Canada (aged 16-24 years) have the highest rates of mental health and addiction concerns across all age groups and the most unmet health care needs [1]. Mental health or substance use concerns are experienced by 1 in 5 young people [2]. Significantly, it is during this time in their lives when youth no longer qualify for most child services and must meet eligibility criteria if they need to access adult mental health services [3]. This transition period is associated with higher rates of treatment disengagement [4], illness progression [5], and youth feeling that their voices are not being heard [6,7]. During this transitional time, youth may also experience poorer functioning in their daily lives, homelessness, involvement with justice agencies, and challenges with education and employment [8].

Research from Canada, the United Kingdom, the United States, and Australia demonstrates that siloed institutional structures create an inadequate interface between the child and adult mental health systems [9]. Youth require significant informational and motivational supports during this period, but current service approaches often leave them feeling uninformed and left with little to no help [10]. In fact, numerous studies have noted an absence of transition planning supports with youth altogether [11,12]. Closing critical service gaps for youth at key transition points is a major focus of mental health and institutional policy and strategy [13]. However, in designing supports and services that promote the purposeful and gradual shifting of care from child to adult mental health services, the voice of youth with lived experiences is essential. Centering the preferences, experiences, and knowledge of youth in the interventions and strategies used to support transitions is a necessary step in shifting from disempowering and paternalistic approaches to service planning that respects their right to agency [14]. In particular, a recent systematic review pointed to the lack of research involving a range of transition-age youth and a predominant focus on either children or adults [10].

While a variety of in-person supports and programs have been trialed [15], there is some evidence that modern technology modalities can connect youth to just-in-time information and services during transitions in care [16], align with their preferences [17], and reflect how they use digital tools in their everyday lives [18]. Youth have identified SMS text messaging as a preferred communication tool for support during the transition to adult services generally [19]. Text messaging, also known as short-messaging service (SMS), is among the most frequently used technologies for low-intensity behavioral health interventions that focus on promoting positive actions linked to healthy outcomes [20]. These behavioral prompts are important during the transition period as youth are not only facing information needs but must navigate a complex range of new actions, social relationships, and developmental changes (eg, book an appointment with their new adult mental health clinician on their own). A recent scoping review [21] showed that SMS text message–based interventions are highly acceptable and scalable, with positive findings reported across a range of adolescent mental health service contexts, including 1 reviewed study that focused on key transition points in recovery [22]. A meta-analysis undertaken by Head et al [23] reported interventions adopting SMS text messaging to have an effect size of *d*=0.33 on health behaviors. Given the easy reach and scalability of SMS text message interventions, even modest effects can have significant public health impacts [24]. SMS text message–based interventions could play a complementary role in helping youth adopt healthy behaviors and strategies associated with positive transitions in ways traditional paper-based or in-person services cannot. However, there is a lack of knowledge regarding youth preferences for content and technology functionality of SMS text message interventions.

Many digital interventions aimed at addressing health behaviors do not report on what evidence or theory underpinned the message content or timing [25], or whether and how a user-centered design approach was used [26]. Intentional and transparent reporting of underpinning theories can better show how interventions could be adapted for local contexts, allow replicability and comparability with other studies, and improve our understanding of how and why the intervention might work for different populations [27]. Two commonly used frameworks in health technology intervention design offer a helpful starting point in developing SMS text message–based interventions for youth transitions. The information-motivation-behavioral skills (IMB) model, widely used in understanding and improving health behavior and specifically applied in studies with various youth populations dealing with chronic diseases [28,29], suggests that clients need to be informed, motivated, and behaviorally skilled to adopt, implement, and maintain positive health behaviors [30]. The persuasive system design (PSD) model has been used extensively to map youth mental health interventions [31,32] and conceptualize features and functionality that might promote engagement and adherence to digital health interventions [33] in 4 main ways. PSD can help intervention designers consider “primary task supports” that might enable users to complete an intended task (eg, tailoring content to different group’s unique needs), “dialogue supports” that can motivate users to stay engaged with the intervention (eg, autoreminders to complete a daily entry), “system credibility supports” that enhance users’ perception that information is dependable (eg, citing a reputable source), and “social supports” that can leverage group influence as a motivator (eg, pooling individual results in a group score).

Informed by the theoretical constructs in the IMB and PSD models, this study aimed to better understand the preferences of transition-age youth to inform future co-design and evaluation of an SMS text message–based intervention. From that co-design perspective, the insights, priorities, and strategies of youth with lived experiences with mental health and substance use should be centered [34]. To this end, this study assessed (1) youth preferences related to the content of potential SMS text messages, (2) the most important features and functionality of the technology system delivering the SMS text messages, and (3) perceived barriers and enablers to staying engaged with an SMS text messaging service over the transition period.

## Methods

### Study Design

We followed guidance for mobile phone–based, brief SMS text message development, aimed at promoting good intervention design mapping before experimental testing [35,36]. This was done by reviewing existing literature, reviewing locally created and relevant resources, applying theory, and getting user feedback on acceptability and relevance early in the design process [27,37]. Following institutional review board approval, we conducted a cross-sectional online survey of a convenience sample of young people. A survey was selected for data collection as it was an efficient way to inform rapid prototyping of a minimal viable product version intervention, that is, a product comprised of features and functionalities that address the most immediate needs of the end user, can attract early adopters, and efficiently validate the initial product idea [38]. Survey-based testing yields similar results, as focus groups with significantly lower burden can be iterated and modified quickly to test subtle variations [39]. Previous research has successfully used convenience sampling within the context of e-mental health, including a Canadian study conducted using a web-based survey with youth, recruited from the general population based on their experiences with web-based and traditional mental health resources [40].

### Ethical Considerations

This study has been funded by the Canadian Institute of Health Research (CIHR) grant (#424885) and has been approved by the IWK Health Research Ethics Board (IWK REB #1027212). Electronic informed consent was obtained from all participants through REDCap (Research and Electronic Data Capture; Vanderbilt University) before participation. No parental consent was required for youth aged between 16 and 18 years. Participation in the study was voluntary and participants were given a CAD $15 (approximately US $10.85) gift card for their time, after completing the survey. All information gathered about the participants was kept private and confidential. Data were deidentified before analysis.

### Setting and Population

Transition-age youth encompasses a broad demographic spanning from older adolescence to young adulthood, although not all countries apply the same age bracket. In some studies, this age goes as low as 12 years and as high as 29 years, but typically, transition-age youth are those in the 15-26 years age range. Youth living in Canada aged 16 to 26 years, who reported accessing mental health services in Canada within the past 5 years and were able to complete the survey in English, were eligible. As an exploratory study within a larger multiphased project to codevelop a prototype intervention with youth, we aimed to recruit 100 participants for initial input through the survey.

### Survey Development

The research team mapped recent and relevant research conducted with youth around needs and preferences for service supports during the transition period [12,41-43], local transition service program content (the Stay Connected Mental Health Project) informed by youth engagement [44], clinical practice guidelines [45], and national and international position papers and reports on quality transition practices [46]. A rapid content analysis of those key resources [47] generated a list of unique items for potential SMS text message content (20 items), a list of technology features (21 items), a list of barriers (8 items), and a list of enablers (8 items). In total, 3 youths reviewed the survey items for face and content validity, and revisions for clarity were incorporated. The survey consisted of 4 parts: demographics, content of SMS text messages, features and functionality, and barriers and enablers to engagement.

#### Demographics

Demographic information collected included age, gender, cultural groups most identified with, current use of SMS text messaging, current enrollment in school, current working situation, current living situation, current access to mental health services, and age when mental health services were first accessed.

#### Content of SMS Text Messages

The 20 potential content topics were organized into “informational” (eg, description of the role of parents during the transition period), “motivational” (eg, stories from other youth who have completed the transition process), and “behavioral” (eg, reminders and notifications of key transition activities) domains. Youth were asked to rate the level of importance for each item using a 7-point Likert scale (1=not important at all, 2=not important, 3=slightly not important, 4=neutral, 5=slightly important, 6=important, and 7=extremely important).

#### Features and Functionality

The 21 potential technology and media features and functionalities (eg, number of SMS text messages to receive, ability to tailor texts, and including links to videos) that could be leveraged in an SMS text message intervention were categorized according to the 4 PSD categories. Youth were asked to rate the level of importance for each item using the same 7-point Likert scale used for content messages.

#### Barriers and Enablers

In total, 3 questionnaire items explored perceived barriers and enablers to youth engagement with the SMS text message intervention. Specifically, 2 items used a “select top response” option and 1 was an open-ended question.

### Recruitment and Enrollment

Recruitment occurred during the COVID-19 pandemic, so a multichannel online recruitment strategy was used, including paid and organic social media and targeted promotion by partner health agencies. Recruitment materials were developed with inputs from 4 youths aged between 15 and 21 years. Interested participants were directed to the study website, which listed inclusion criteria, the purpose of the study, and contact information for the study coordinator to answer any questions. Youth were required to review an information sheet and confirm understanding before being able to access the survey items securely through REDCap. REDCap is a secure application that provides an interface for data entry, audit trails for tracking data export and analysis, automated data export to various statistical software, and the ability to import data from outside sources [48]. Consent was obtained from all participants. Data were collected between November 2021 and March 2022, and the participants took approximately 15 minutes to complete the survey. A gift card honorarium (CAD $15 [approximately US $10.85]) was sent to all participants.

### Statistical Analysis

IBM SPSS Statistics [49] was used, and analysis occurred in 3 basic steps. First, descriptive statistics were used to characterize the full sample. Second, we identified the cutoff for levels of consensus (Table 1) informed by other published consensus matrices [39,50]. Original response options “not important at all” and “not important” were combined into a “not important” category, and original options “extremely important” and “important” were combined to represent an “important” comparison group. Locally, service users in the age range of 16-19 years are served by the child system, and those aged 20 years and older are served by the adult system. Since the end goal of the study was to inform local development of an SMS text messaging service, we applied these age ranges for descriptive analysis. Finally, we used chi-square tests to examine whether group differences in age (18 years or younger and 18 years or older), gender (men, women, prefer to self-describe, and prefer not to say), and ethnicity (non-White and White) were statistically significant at the *P*<.05 level. Where assumptions of normal distribution were violated (ie, skewness values exceeded −3 to +3 and kurtosis values exceeded −10 to +10) [51], Mann-Whitney *U* tests and 1-way nonparametric ANOVA (Kruskal-Wallis test) were used.

**Table 1:** Level of importance consensus matrix.

Priority level	High consensus	Moderate consensus	Low consensus	No consensus
Important	≥70% reporting “extremely important” or “important”	60%-69% reporting “extremely important” or “important”	50%-59% reporting “extremely important” or “important”	<50% reporting “extremely important” or “important”
Not important	≥70% reporting “not important at all” or “not important”	60%-69% “not important at all” or “not important”	50%-59% reporting “not important at all” or “not important”	<50% reporting “not important at all” or “not important”

## Results

### Overview

**Figure 1:**
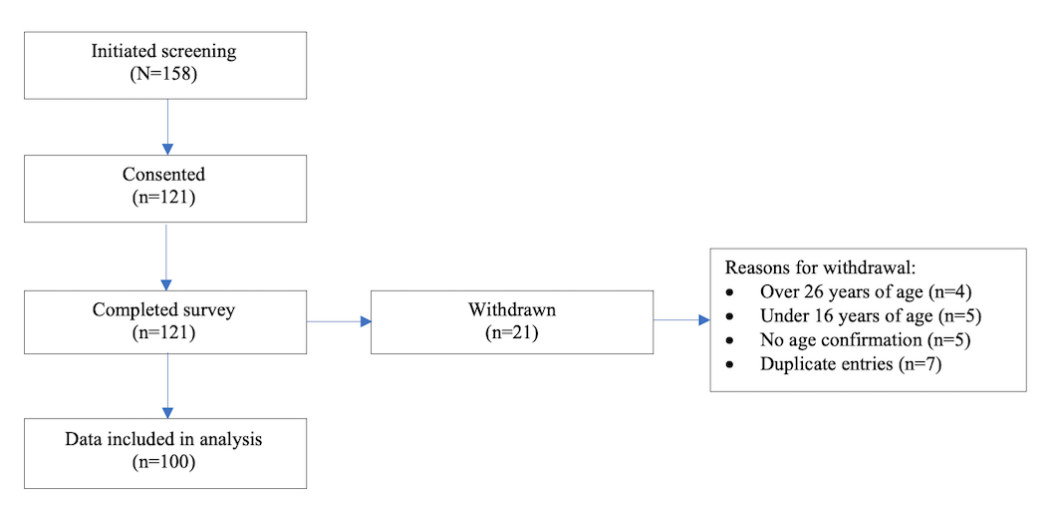
e-Delphi study participant recruitment and enrollment flow diagram.

An overview of recruitment and enrollment is presented in Figure 1. A total of 158 participants visited our study web page and initiated screening. Of that, 77% (121/158) consented to participate. Out of 158 participants, 14% (21) were withdrawn from the study for being outside the eligible age range or for duplicate entries. Data from 100 youths were included in the analysis.

### Demographics

A total of 100 participants, predominantly aged between 20 and 26 years (n=59, 59%), women (n=65, 65%), and who had first accessed mental health services when they were between 13 and 19 years of age (n=60, 60%; Table 2), were selected. A significant portion of participants identified as 2SLGBTQIA+ (two-spirit, lesbian, gay, bisexual, transgender, queer/questioning, intersex, asexual/agender, and additional people who identify as part of sexual and gender diverse communities; 44/100, 44%) and were from non-White ethnic groups (47/100, 47%). More than half of participants reported that they were living at home with their parents (62/100, 62%) and accessing mental health services at the time of completing the survey (76/100, 76%). Almost all participants (90/100, 90%) reported being daily SMS text message users.

**Table 2:** Baseline demographic characteristics.

Characteristics		Participants (n=100), n (%)
**Age group (years)**		
	16-19	41 (41)
	20-26	59 (59)
**Gender**		
	Woman	65 (65)
	Man	18 (18)
	Prefer to self-describe (eg, agender and nonbinary)	16 (16)
	Prefer not to say	1 (1)
**Ethnicity**		
	South Asian (East Indian, Pakistani, Sri Lankan, or Bangladeshi)	16 (16)
	East Asian (Chinese, Japanese, Korean, or Polynesian)	15 (15)
	Indigenous (First Nation, Inuit, or Metis)	6 (6)
	Southeast Asian (Vietnamese, Cambodian, Laotian, or Thai)	4 (4)
	Middle Eastern (Egyptian or Lebanese)	3 (3)
	West Asian (Iranian or Afghan)	2 (20
	African Nova Scotian or African-Canadian, African-American, or Caribbean	1 (1)
	White	42 (42)
	Prefer not to answer or not reported	11 (11)
**Diversity**		
	2SLGBTQIA+^a^	44 (44)
	Differently abled	14 (14)
	Recent immigrant (immigrated to Canada or Nova Scotia in the last 5 years)	1 (1)
**Education**		
	Enrolled in school	63 (63)
	Not enrolled in school	37 (37)
**Employment**		
	Employed	60 (60)
	Unemployed	40 (40)
**Living situation**		
	Living with parents	62 (62)
	Rent	30 (30)
	Own	3 (3)
	Other	5 (5)
**Current use of mental health services**		
	Currently accessing	76 (76)
	Currently not accessing	24 (24)
**Age of first mental health service access**		
	12 years or younger	23 (23)
	13-19 years	60 (60)
	20-25 years	13 (13)
	Not reported	4 (4)
**Current use of SMS text messaging**		
	Daily	90 (90)
	Weekly	7 (7)
	Rarely	3 (3)

^a^2SLGBTQIA+: two-spirit, lesbian, gay, bisexual, transgender, queer/questioning, intersex, asexual/agender, and additional people who identify as part of sexual and gender diverse communities.

### Preferred Content of Messages

Based on the rating matrix (Table 1), a high level of consensus around importance was reported in 45% (9/20) of content items (Table 3). The content item with the highest level of consensus (88/100, 88%) was “the description of what to expect in your first adult mental health service appointment.” Other information-focused items that met the high consensus threshold were “contact information for who youth can speak to” (79/100, 79%), “a list of potential benefits for completing steps in the transition process” (74/100, 74%), and “a description of the role of health care providers in the transition process” (73/100, 73%). Only 2 of the 8 motivation-focused content items met the high consensus threshold: “affirming feelings of frustration or disappointment in having to make the change” (77/100, 77%) and “reminders of available self-care supports youth can access” (70/100, 70%). All 3 of the behavior-focused items met the high level of consensus threshold: “link to the location or address of the adult community mental health clinic youth will be visiting” (81/100, 81%), “reminders or notifications about upcoming dates or actions” (78/100, 78%), and “demonstration of how to prepare for the first adult appointment” (76/100, 76%). Alternatively, 15% (3/20) of all content items did not even achieve a low level of consensus on importance: “stories from other youth who have completed the transition process” (49/100, 49%), “statistics on the positive impacts of following through on transition goals” (45/100, 45%), and “statistics on the negative impacts of not following through on transition goals” (36/100, 36%).

Preliminary inspection of data prior to chi-square analysis revealed assumptions of normal distribution were violated for 55% (11/20) of content items on the survey, with skewness for rating items as important. Mann-Whitney *U* and Kruskal-Wallis tests were used in place of chi-square in those instances. No statistically significant relationships between age group and individual content item ratings of importance were identified (all *P*>.05). Statistically significant differences were observed between ethnic groups and 2 individual content items. This included “seeing statistics on the negative impacts of not following through on transition goals” (behavior-focused item; χ^2^_2_=6.7; *P*=.04) and “interactive activities for dealing with transition anxiety” (motivation-focused item; U=918; *P*=.006). In both instances, non-White respondents attributed higher importance compared with participants who self-reported being White. Only 1 item, “a list of potential benefits for completing steps in the transition process” (information-focused item), showed a statistically significant difference (Kruskal-Wallis *H*(2)=6.455, *P*=.04) based on gender groups, with women attributing higher importance to this item compared with men and participants with other self-described genders.

**Table 3:** Perceived importance for content items according to information-motivation-behavioral skills model dimensions.

Domains and items			Level of important and participants (n=100), n								
			1^a^		2^a^		3^a^		4^a^		5^a^
**Information^b^**											
	Description of what to expect in your first adult appointment^c^	88 (88)		9 (9)		1 (1)		0 (0)		2 (2)	
	Contact information for who you can speak to if you have questions^c^	79 (79)		16 (16)		4 (4)		1 (1)		0 (0)	
	List of potential benefits for completing steps in the transition process^c^	74 (74)		17 (17)		6 (6)		2 (2)		1 (1)	
	Description of the role of health care providers in the transition^c^	73 (73)		23 (23)		4 (4)		0 (0)		0 (0)	
	A picture that outlines the steps needed for a successful transition	60 (60)		24 (24)		4 (4)		7 (7)		5 (5)	
	General tips and links to online mental health supports	55 (55)		27 (27)		10 (10)		3 (3)		5 (5)	
	Links to fillable forms where you can set goals or create to-do lists	55 (55)		28 (28)		8 (8)		4 (4)		5 (5)	
	List of possible negative impacts of not completing transition steps	53 (53)		24 (24)		15 (15)		6 (6)		2 (2)	
	Description of the role of parents or caregivers in the transition process	51 (51)		24 (24)		11 (11)		9 (9)		5 (5)	
**Motivation^b^**											
	Affirming feelings of frustration or disappointment in the change^c^	77 (77)		13 (13)		9 (9)		0 (0)		1 (1)	
	Reminder of available self-care supports you can access^c^	70 (70)		19 (19)		7 (7)		1 (1)		3 (3)	
	Interactive activities for dealing with transition anxiety	65 (65)		29 (29)		5 (5)		0 (0)		1 (1)	
	Reminder of community programs and supports you can access	61 (61)		25 (25)		9 (9)		3 (3)		2 (2)	
	Reminder of your own goals for the transition process	59 (59)		30 (30)		9 (9)		2 (2)		0 (0)	
	Stories from other youth who have completed the transition process	49 (49)		29 (29)		16 (16)		4 (4)		2 (2)	
	Statistics on the positive impacts of following through on goals	45 (45)		25 (25)		18 (18)		9 (9)		3 (3)	
	Statistics on the negative impacts of not following through on goals	36 (36)		27 (27)		22 (22)		10 (10)		5 (5)	
**Behavior^b^**											
	Location or address of the adult community mental health clinic^c^	81 (81)		15 (15)		3 (3)		1 (1)		0 (0)	
	Reminders or notifications about upcoming dates or actions^c^	78 (78)		18 (18)		3 (3)		0 (0)		1 (1)	
	Demonstration of how to prepare for the first appointment^c^	76 (76)		16 (16)		5 (5)		2 (2)		1 (1)	

^a^1=extremely important or important; 2=slightly important; 3=neutral; 4=slightly not important; 5=not important at all or not important.

^b^Items are ordered from highest to lowest levels of agreement on importance with the first and last 2 categories of the Likert scale collapsed.

^c^Met the consensus threshold.

### Preferred Features and Functionality

A high level of consensus on importance ratings was observed in 19% (4/21) of technical feature and functionality items (Table 4). Only 1 primary task support–focused item reached the high consensus threshold: “information is tailored to the specific mental health condition of the end user” (71/100, 71%). All 3 system-credibility–focused items met threshold levels of high consensus: “ability to stop or start texts at any point” (82/100, 82%), “information sounding truthful, fair, and unbiased” (78/100, 78%), and “evidence that texts are coming from a trusted health care center” (71/100, 71%). None of the dialogue or social support–focused items met the high level of consensus thresholds.

**Table 4:** Perceived importance of technical features and functionality according to persuasive system design dimensions.

Domains and items		Level of importance and number of participants (n=100), n				
		1^a^	2^a^	3^a^	4^a^	5^a^
**Primary task support^b^**						
	Information is tailored to specific mental health condition^c^	71 (71)	20 (20)	7 (7)	2 (2)	0 (0)
	Options to explore more about the topic by clicking on links	63 (63)	24 (24)	10 (10)	3 (3)	0 (0)
	Personalized texts that reference you by name	63 (63)	15 (15)	13 (13)	1 (1)	8 (8)
	Information is tailored to the age of the end user	63 (63)	16 (16)	14 (14)	3 (3)	4 (4)
	Quizzes and checklists you complete to see how you are doing	60 (60)	26 (26)	9 (9)	1 (1)	4 (4)
	Receiving the texts at the same time of day each time	54 (54)	24 (24)	10 (10)	4 (4)	8 (8)
	SMS text messages written at a basic level (eg, grade 8 reading level)	51 (51)	16 (16)	18 (18)	4 (4)	11 (11)
	Receiving less than 10 texts in total	47 (47)	17 (17)	25 (25)	4 (4)	7 (7)
	"Single messages no longer than 160 characters each"	45 (45)	21 (21)	23 (23)	5 (5)	6 (6)
	Receiving SMS text messages between 3 and 6 months	34 (34)	27 (27)	27 (27)	2 (2)	10 (10)
	Receiving SMS text messages for 3 months or less in total	22 (22)	20 (20)	35 (35)	12 (12)	11 (11)
	Receiving more than 10 texts in total	13 (13)	16 (16)	46 (46)	9 (9)	16 (16)
**Dialogue support^b^**						
	Reminders to complete specific transition plan activities	66 (66)	26 (26)	6 (6)	0 (0)	2 (2)
	Hopeful or encouraging language in the texts (eg, “You are not alone”)	64 (64)	19 (19)	8 (8)	7 (7)	2 (2)
	Embedded links to videos, websites, or documents	49 (49)	24 (24)	19 (19)	4 (4)	4 (4)
	Automated bounce-back messages of encouragement (eg, “Great job!”)	49 (49)	26 (26)	12 (12)	4 (4)	9 (9)
**System credibility support^b^**						
	Ability to stop or start texts at any point^c^	81 (81)	16 (16)	2 (2)	0 (0)	1 (1)
	Information sounding truthful, fair, and unbiased^c^	78 (78)	18 (18)	2 (2)	2 (2)	0 (0)
	Evidence that texts are coming from a trusted health care center^c^	71 (71)	21 (21)	5 (5)	2 (2)	1 (1)
**Social support^b^**						
	Ability to share a link to the program with others	46 (46)	30 (30)	12 (12)	4 (4)	8 (8)
	"Seeing how many youths have signed up for the SMS text messages"	24 (24)	16 (16)	22 (22)	16 (16)	22 (22)

^a^1=extremely important or important; 2=slightly important; 3=neutral; 4=slightly not important; 5=not important at all or not important.

^b^Items are ordered from highest to lowest levels of agreement on importance with the first and last 2 categories of the Likert scale collapsed.

^c^Met the consensus threshold.

Preliminary inspection of the data revealed assumptions of normal distribution were violated for 24% (5/21) of technology feature and functionality items. No significant differences in ratings of importance for technology feature items were observed based on age groups (all *P*>.05). Statistically significant differences based on ethnicity were observed for 2 items: "seeing how many youths have signed up for the SMS text messages" (social support item; χ^2^_2_=7.8; *P*=.02) and "single messages no longer than 60 characters each" (primary task support item; χ^2^_2_=6.2; *P*=.045). In both instances, non-White respondents attributed higher importance compared with participants who self-reported being White. Finally, “a single message no longer than 60 characters each” (primary task support item) also showed a statistically significant difference (χ^2^_4_=13.6; *P*=.009) based on gender groups, with women attributing higher importance to this item compared with men and participants with other self-described genders.

### Barriers and Enablers

The most significant reported enablers to engaging with an SMS text message intervention for youth during the transition period to adult mental health services were SMS text messages being personalized (27/100, 27%), texts grabbing their attention (20/100, 20%), and receiving a reasonable number of texts (15/100, 15%; Table 5 ). Another 12% (n=12) of participants thought it was important that the texts were short and straight to the point. Only 4% (n=4) thought including links to videos or visuals would help them engage with an intervention.

The largest barriers to engaging with an SMS text message intervention included: SMS text messages not grabbing their attention (18/100, 18%), SMS text messages being too long (16/100, 16%), and receiving too many SMS text messages (15/100, 15%) in total. In addition, some youth stated that if texts were repeating information they were receiving elsewhere, it would be a barrier (15/100, 15%). In total, 12% (n=12) of the participants thought that the SMS text messages not being from a credible source would be a barrier. Alternating the time of day when SMS text messages were sent was less of a barrier (8/100, 8%).

**Table 5:** Frequency of reported barriers and enablers to text message service engagement among youth.

Items			Respondents (n=100), n (%)
**Enablers**			
	Texts are personalized	27 (27)	
	Texts are interesting or grab my attention	20 (20)	
	I receive a reasonable number of texts	15 (15)	
	Obvious that texts are form a credible source	13 (13)	
	Texts are short and to the point	12 (12)	
	Texts are not too long or too short	8 (8)	
	Texts include links to videos or visuals	4 (4)	
	None of the above	1 (1)	
**Barriers**			
	Texts don’t grab my attention	18 (18)	
	Texts are too long	16 (16)	
	Getting the same info from elsewhere	15 (15)	
	Receive too many texts	15 (15)	
	Texts aren’t from a credible source	12 (12)	
	Not checking the texts I receive	10 (10)	
	Texts are sent at odd times	8 (8)	
	None of the above	6 (6)	

## Discussion

### Principal Findings

Co-design research is a promising methodology for increasing youth engagement in digital health and well-being interventions [52]. The necessity of improving intervention design is evidenced by the vast majority of digital mental health interventions “in the real world” still reporting low engagement and high attrition [53,54]. Our study is the first study to apply theory to systematically document youth preferences for an SMS text message intervention, aimed at supporting the transition to adult mental health services. This study also revealed aspects of the intervention design that can be improved to address issues with volume, timing, and content and to further personalize the experience.

A vast majority of youth in our study were daily users of SMS text messaging, but they were highly diverse in almost every other area of their lives (employment, school, living arrangements, and age of first access to mental health services). Yet, across that diverse population, there were clear user preferences for certain kinds of message content over others. While few information-focused items reached high-consensus levels for importance, this does not mean that informational resources are not valued. It could indicate that more exploratory work is needed to identify different kinds of informational supports youth are looking for that were not captured in our study. Content items with high consensus on importance (eg, description of what to expect at a first adult appointment, contact info, location or address of the adult community mental health clinic, and reminders or notifications about upcoming dates or actions) align with previous research that reports youth often engage in texting as a form of microsocial planning (ie, communicating details about where and when to meet, homework, schedules, and other logistics) [17]. If this is the case, then an SMS text message intervention for transitions that delivers more planning-focused information than knowledge-gathering information (eg, links to external resources, stories from other youth, and statistics on expected results) may be viewed as more valuable. While this study identified preferred content topics that can inform a rapid prototype development, the reasons why those topics were valued in that way, are valid areas for future research.

While similarities across youth subpopulations in our study bode well for developers interested in standard topical messaging across different contexts, more research is needed to understand how dimensions of intersectionality influence youth preferences in mobile mental health interventions. Recent research among adults suggests that differences may be more subtle and would require larger sample sizes to tease apart [39]. It may be that age, gender, and ethnicity are not the primary dimensions on which to tailor message content during the transition period to adult mental health services but that other determinants of health (eg, housing status, geographic distance to the health care provider, and employment status), personal characteristics (eg, personal values), or specific mental health diagnosis could be. Using behavioral theory to design effective SMS text messaging interventions is an ongoing challenge and superficial use of behavior change theory is often an obstacle to effective intervention design [36].

Our challenge was how to map “key ingredients” [55] of transition program technology features found in the literature and local resources onto PSD domains and how to measure the level of consensus around whether youth saw these items as important enough to be included in a future intervention. In line with other research using the PSD framework to explore SMS text message–based health interventions [56], our study found youth had no ideal combination of techniques or principles they viewed as vital. There is no clear direction on a set of persuasive design features that will combine in a simple additive fashion to make an intervention better. That said, consistent with mental health SMS text messaging research [57], personalization was reported as the most significant enabler to engagement. For preferences that are less strongly held, the program can collect ongoing ratings or engagement data to inform passive, gradual tailoring, without complete exclusion of a content type [57]. There is limited guidance in the academic literature on approaches and strategies to conceptualize and translate these theoretical constructs into real-world digital mental health intervention content and features. The findings of this study, including key barriers and facilitators, will inform a more intentional and focused prototype development phase, but the extent to which the future intervention will elicit the intended informational, motivational, or behavioral outcomes and improve the transition to adult mental health services remains uncertain [58]. Our results are consistent with much of the health communications literature in terms of outcomes indicating that preferences research is a useful preliminary step in intervention development.

### Implications for Research and Practice

Youth transitioning to adult mental health services identified personalized and practical information included in an SMS text message intervention as the key enabler to intervention engagement. This finding aligns with other research on tailoring messages because personal context increases receptivity, memory for messages, self-relevance, and self-referential processing of information [59]. This research is important as it could help service providers and health care organizations create and trial novel transition interventions in response to gaps and the need for new approaches. The findings from this study will help us develop a prototype intervention and plan a controlled trial. We will be able to examine potential pathways that may explain how the intervention supports different kinds of youth behavior change during the transition period.

### Limitations and Strengths

Four main limitations should be noted. First, we did not restrict the availability of the survey to people who were currently transitioning to adult mental health services. It is possible that this wider population may be thinking ahead to transitions or reflecting on a recent transition period and may have different message preferences than a sample currently experiencing transition challenges. Second, external validity should be considered when interpreting results as we did not estimate the necessary sample size for this study. Since the study was not evaluating treatment effects and was one early exploratory strategy to solicit youth opinions as part of a multicomponent design project, our sample was relatively small. Third, we used a 70% threshold for the levels of consensus. While a threshold percentage is not always provided a priori in most Delphi studies, the range reported as an accepted consensus is very wide (50%-97%) [60]. Still, our threshold was slightly lower than the more frequently used 75% [61]. Pragmatically, we wanted to rapidly identify priority features and functionality that would be later refined with youth during a design workshop. Finally, a minor limitation of this study is that in some countries (eg, the United States) there are limitations to direct SMS text messaging of youth aged 18 years or younger unless their parent or guardian is included in the communication as well. This may have a small impact on the generalizability of this study.

Despite these limitations, co-design strategies that help identify and disseminate youth perspectives on intervention design are needed, and this study makes a unique contribution to a small body of current evidence related to transition-focused technology solutions. However, findings from this study should be considered preliminary until more research can be done.

## Abbreviations

2SLGBTQIA+: two-spirit, lesbian, gay, bisexual, transgender, queer/questioning, intersex, asexual/agender, and additional people who identify as part of sexual and gender diverse communities
CIHR: Canadian Institute of Health Research
IMB: information-motivation-behavioral skills
PSD: persuasive system design
REDCap: Research and Electronic Data Capture

## Data Availability

The data sets generated and/or analyzed during this study as well as the survey are available from the corresponding author on reasonable request.
